# Marine Reptiles

**DOI:** 10.1371/journal.pone.0027373

**Published:** 2011-11-08

**Authors:** Arne Redsted Rasmussen, John C. Murphy, Medy Ompi, J. Whitfield Gibbons, Peter Uetz

**Affiliations:** 1 School of Conservation, The Royal Danish Academy of Fine Arts, Copenhagen, Denmark; 2 Division of Amphibians and Reptiles, Field Museum of Natural History, Chicago, Illinois, United States of America; 3 Marine Biology Laboratory, Faculty of Fisheries and Marine Sciences, Sam Ratulangi University, Manado, North Sulawesi, Indonesia; 4 Savannah River Ecology Lab, University of Georgia, Aiken, South Carolina, United States of America; 5 Center for the Study of Biological Complexity, Virginia Commonwealth University, Richmond, Virginia, United States of America; University of Western Australia, Australia

Of the more than 12,000 species and subspecies of extant reptiles, about 100 have re-entered the ocean. Among them are seven species of sea turtles and about 80 species and subspecies of sea snakes, as well as a few other species that are occasionally or regularly found in brackish waters, including various other snakes, the saltwater crocodile, and the marine iguana of the Galapagos Islands. The largest group of marine reptiles, the sea snakes, occur in the tropical and subtropical waters of the Indian and Pacific Oceans from the east coast of Africa to the Gulf of Panama. They inhabit shallow waters along coasts, around islands and coral reefs, river mouths and travel into rivers more than 150 km away from the open ocean. A single species has been found more than 1000 km up rivers. Some have also been found in lakes. The taxonomic status of the sea snakes is still under review and no general agreement exists at the moment. The effects of the exploitation on sea snakes have been investigated in the Philippines and Australia but are almost unknown from other areas. Investigations indicate that some populations are already extinct and others are in danger of extinction in various parts of Asia. All sea turtles are endangered except one. The marine iguana of the Galapagos Islands remains vulnerable due to its limited range. Brackish water snakes are closely associated with mangrove forests and as such are subject to deforestation and coastal development schemes that result in habitat loss. In addition, some are collected for their skins. While none of the coastal species are considered in danger of extinction at the present time, many are data deficient.

## Introduction

Reptiles are the most diverse terrestrial vertebrates with about 12,000 described forms, including about 9,350 currently recognized species and about 3,000 subspecies [1].

About 260 million years ago reptiles evolved from aquatic amphibians and by the Jurassic (150–200 myr) modern reptiles had appeared. However, only a few reptile groups re-entered the oceans, primarily sea snakes (elapids related to cobras and kraits), and sea turtles. All major reptile groups, i.e. the snakes, lizards, turtles, and crocodiles, have at least a few members that enter marine habitats even though they may have never completely adapted to a life in the open sea. Here we give an overview of those reptiles that are found exclusively or at least occasionally in the oceans.

## Sea Turtles

**Figure 1:**
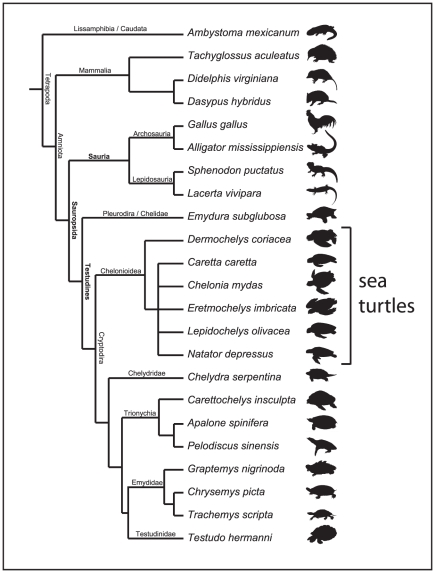
Phylogeny of sea turtles. (A) Phylogenetic relationships of amniotes with the position of sea turtles relative to other vertebrates. Modified after [124].

**Figure 2:**
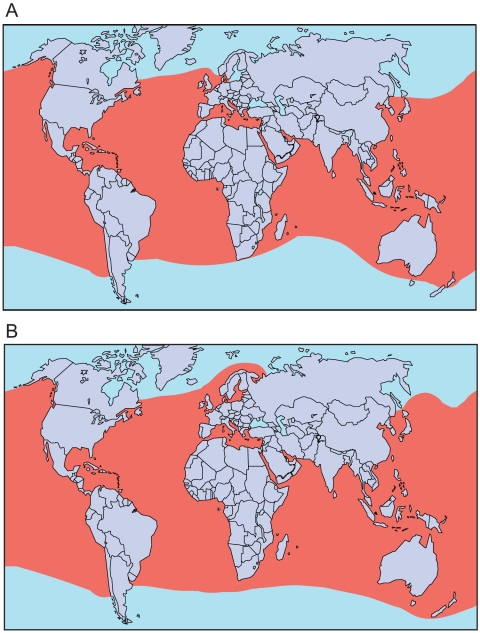
Distribution of sea turtles. (A) Cheloniidae. (B) Dermochelyidae. From [125]. Detailed maps for individual species can be found in [126].

Sea turtles arose about 100 million years ago from terrestrial or fresh-water turtles (**Figure 1**) [2]. Currently only 7 species are extant (**Table 1**) although certain authors list *Chelonia mydas agassizi* as an eighth valid species (e.g. [3]). Sea turtles are found primarily along tropical coasts (**Figure 2**). However, some are also well-known for their long journeys across the oceans. Most species nest along the coasts of Central and South America or in the Caribbean, although some species occasionally travel as far north as Scandinavia.

**Table 1:** All species of sea turtles.

Species	Common name	IUCN Red List status*
*Caretta caretta*	Loggerhead	Endangered
*Chelonia mydas*	Green Turtle	Endangered
*Dermochelys coriacea*	Leatherback	Critically Endangered
*Eretmochelys imbricata*	Hawksbill	Critically Endangered
*Lepidochelys kempii*	Atlantic Ridley	Critically Endangered
*Lepidochelys olivacea*	Pacific Ridley	Vulnerable
*Natator depressus*	Flatback Turtle	? (data deficient)

*IUCN Red List data from http://www.iucnredlist.org/.

Like many other reptiles, most if not all sea turtles seem to use temperature-dependent sex determination (TSD). TSD has been demonstrated for loggerhead (*Caretta caretta*), green (*Chelonia mydas*), leatherback (*Dermochelys coriacea*) and olive ridley (*Lepidochelys olivacea*) turtles [4]. Since TSD is often sensitive to small changes in temperature, global warming may eventually affect sex ratios in these species and, together with their general vulnerabilities (see below), have a dramatic effect on their reproductive rate and thus long-term survival.

### Migration

Usually, sea turtles travel the seas on their own. However, during the nesting season they head towards their home beaches and may form large groups of turtles traveling together, even if they maintain distances of up to several hundred meters between individuals. The ability of sea turtles to find their original nesting sites has spurred considerable interest. Only recently it has been shown that sea turtles use geomagnetic sensing to orient themselves [5], [6]. While sea turtles appear to return to a certain geographic region, they not necessarily return to a specific. However, Kemp's Ridley does nest on only one location in Mexico, so it is especially endangered. It remains unclear though how precise other turtles are in returning [5], [7], [8].

### Conservation

Most sea turtles are endangered or even critically endangered. The Olive Ridley Turtle (*Lepidochelys olivacea*) is vulnerable and for the Flatback Turtle (*Natator depressus*) there is simply not enough data, although in previous years it had been classified as “vulnerable” too. The IUCN Red List says about the Green Turtle (*Chelonia mydas*):

“Analysis of historic and recent published accounts indicate extensive subpopulation declines in all major ocean basins over the last three generations as a result of overexploitation of eggs and adult females at nesting beaches, juveniles and adults in foraging areas, and, to a lesser extent, incidental mortality relating to marine fisheries and degradation of marine and nesting habitats. Analyses of subpopulation changes at 32 Index Sites distributed globally show a 48% to 67% decline in the number of mature females nesting annually over the last 3 generations.” [9]

Similar statements are true for all sea turtles: overexploitation and the destruction of nesting sites cause concern for these species, as tropical beaches are also in high demand for touristic reasons and easily exploited by locals.

## Other Turtles Living in Brackish Environments

Of the more than 300 species of turtles only seven are truly marine while about 50 species are fully terrestrial, belonging to the family of tortoises, the Testudinidae. The majority of the remaining species, and most of the world's turtles, are aquatic or semi-aquatic freshwater species. However, a few are associated with estuarine and other brackish water habitats in an environment that is neither marine nor fresh water (**Table 2**).

**Table 2:** Turtles in brackish waters.

Genus	number of all species	brackish species
*Malaclemys*	1	1
*Batagur*	6	3
*Carettochelys*	1	1
*Pelochelys*	3	1

Included among the species that spend a portion or all of the year in estuarine habitats are the mangrove terrapins (*Batagur affinis* and *B. baska*) of south-east Asia and India, as well as the pig-nosed turtles (*Carettochelys* sp.) in southern New Guinea. Painted terrapins (*Batagur borneoensis*) of south-east Asia characteristically spend an even greater portion of their life cycle in estuarine and brackish waters, even laying their eggs on oceanfront beaches in the same areas as sea turtles. A few additional species of turtles, such as the giant softshell (*Pelochelys cantori*) of Asia, will enter estuarine brackish waters and even into full saltwater habitats temporarily, but the majority of their range includes freshwater habitats.

**Figure 3:**
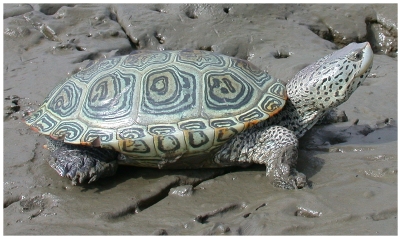
*Malaclemys terrapin*. This species is the only terrestrial turtle with significant adaptions to coastal habitats. Photo courtesy of J.D. Willson, by permission.

The only exclusively brackish water turtle in the world is the diamondback terrapin *(Malaclemys terrapin,* **Figure 3** *)*, which is endemic to tidal creeks and salt marshes as well as the brackish portions of estuaries of the Atlantic and Gulf coasts in the United States, from Cape Cod to Texas. Diamondback terrapins in Florida are found in mangrove swamps. Two species of *Batagur* in Asia and the painted terrapin in Malaysia also occupy mangrove swamps. All of the species found in mangrove swamps also inhabit other brackish waters of coastal areas and most are associated to some degree with river estuaries, and the Asian species commonly enter freshwater systems as well. None of the turtle species in South America, Africa, or Europe has an affinity for brackish water conditions characteristic of the species noted above. However, many species of freshwater turtles that live in coastal areas around the world are known to enter brackish waters on occasion. The diamondback terrapin has a functional salt gland and can live indefinitely in fresh water or sea water. Other estuarine turtles have not been reported to have well developed salt glands comparable to those in the diamondback terrapin.

All turtle species that are brackish water inhabitants face severe conservation threats in all or part of their range because of the numerous environmental impacts on coastal systems throughout the world. Habitat degradation from pollution, sediment runoff, and other consequences of overdevelopment as well as mortality as targeted commercial species or as bycatch from the fisheries industry take an increasingly greater toll on these species.

## Marine Crocodiles

**Figure 4:**
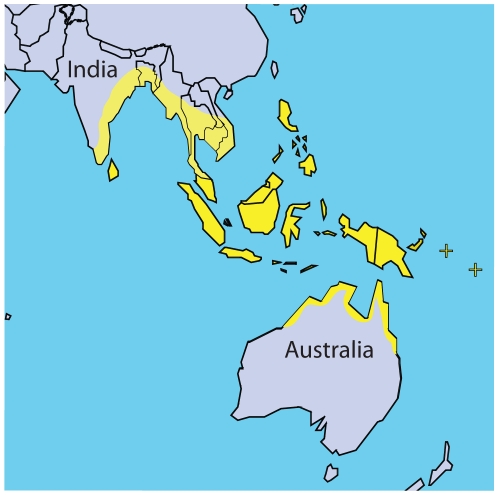
Distribution of the saltwater crocodile, *Crocodylus porosus*. The range is shown in yellow. “+” symbols represent the Pacific islands that are also inhabited by this species, including the Solomon islands and Vanuatu. Adapted from http://www.flmnh.ufl.edu/cnhc/cst_cpor_dh_map.htm.

None of the currently 23 species of crocodile is truly marine (*Crocodylus raninus* has been revalidated as 24th species only recently but this has not been universally accepted). However, at least one species, the Saltwater or “Estuary” Crocodile (*Crocodylus porosus*) is regularly found in brackish waters [10] of south-east Asia and Australia (**Figure 4**). Other crocodiles have been found in tidal waters, such as *Crocodylus johnstoni* or *C. acutus*, but *C. porosus* is the only one that does show some adaptations to salt water [11], [12].

The saltwater crocodile is the largest living crocodile and thus the largest living reptile, reaching a total length of more than 6 meters [13]. The species seems to be most closely related to *C. siamensis* which is not particularly associated with brackish waters [14].

The saltwater crocodile has a high tolerance for salinity, being found in brackish water around coastal areas and in rivers. However, it is also present in freshwater rivers and swamps. Movement between different habitats occurs between the dry and wet season, and as a result of social status: juveniles are raised in freshwater areas, but eventually sub-adult crocodiles are usually forced out of these areas (used for breeding by dominant, territorial adults), into more marginal and saline areas. Subordinate animals unable to establish a territory in a tidal river system are either killed or forced out into the sea where they move around the coast in search of another river system. In recent years in northern Australia, saltwater crocodile populations in some areas have recovered to such an extent that increasing numbers are being forced further upstream into marginal habitat.

Crocodiles use their lingual salt glands to secrete excess salt ions [15]. The morphology of these salt-secreting glands is highly conserved [16]. These tissues are typified by their abundance of ion pumps, responsible for the maintenance of cellular electrochemical gradients through the movement of Na+ and K+ ions against their osmotic gradients. *Crocodylus porosus* possesses lingual salt glands which function to remove excess Na+ and Cl– ions accumulated as a consequence of living in a marine environment [15], [17]. However, other crocodiles, such as the Nile crocodile, seem to have similar glands, even though they may be not as active or efficient as those in the saltwater crocodile [18]. Some authors have suggested that such adaptations to sea water are evidence for a marine evolutionary origin of crocodiles [18].

## Sea Snakes

**Figure 5:**
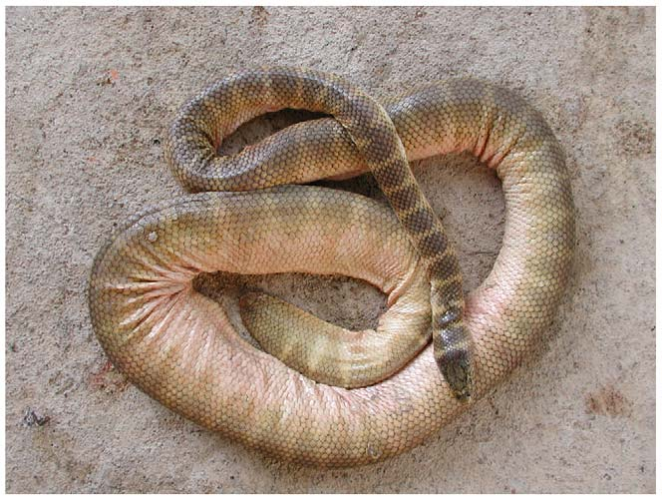
A typical sea snake, *Hydrophis belcheri*. Note the flattened tail as an adaptation to swimming in the open sea.

**Figure 6:**
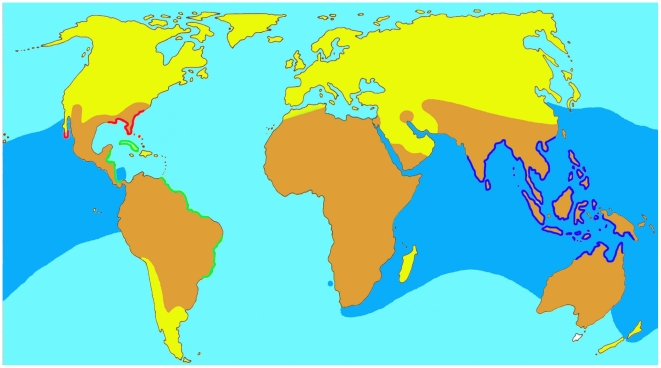
Distribution of marine snakes. Terrestrial distribution represents terrestrial elapids (**brown**), marine distribution represents sea snakes, i.e. the subfamily Hydrophiinae of the Elapidae (**blue**). **Dark blue**: homalopsid snakes along the Asian and Australian coasts. **Red**: North-American Natricidae, **green**: neotropical Dipsadidae.

Besides the sea turtles, the sea snakes are the reptiles that are best adapted to marine environments. The most typical feature of a sea snake is the vertically flattened paddle-like tail, which is not found in any other terrestrial or aquatic snakes (**Figure 5**). Sea snakes occur in the tropical and subtropical waters of the Indian and Pacific Oceans from the east coast of Africa to the Gulf of Panama (**Figure 6**). However, two specimens of *Pelamis platurus* reported from Namibia indicate that the species may be extending its range into the Atlantic Ocean [19]. Most species are concentrated in the Indo-Malayan Archipelago, South China Sea, Indonesia and the Australian region [20]–[29]. Sea snakes inhabit shallow waters along coasts and around islands, river mouths and up rivers for more than 150 km and they have also been found in lakes in Thailand, Cambodia, the Philippines and Rennell Island in the Solomon archipelago [27], [30]–[33]. However, information on precise geographical distribution and abundance for each species is still lacking.

Notably, a few sea snakes are actually freshwater species, such as *Hydrophis semperi, H. sibauensis, H. obscurus,* and *L. crockeri*. However, all evidence indicates that these species have radiated into freshwaters independently from saltwater species [34].

### Sea snake bite

Sea snake bite is the cause of human fatalities. The typical victim is a fisherman handling gape nets or down nets, sorting fish (and sea snakes) on board a trawling boat or dragging a net by wading in muddy coastal waters or in river-mouths. Some sea snakes are gentle, inoffensive creatures that bite only under provocation, but other species are much more aggressive (e.g. *Aipysurus laevis*, *Astrotia stokesii*, *Enhydrina schistosa*, *Hydrophis elegans, H. macdowelli, H. major, H. ornatus and H. ocellatus*) [35]–[39]. As a general rule all sea snakes must be handled with great caution: however, it is worth mentioning that when sea snakes do bite, they do not always inject much or any of their venom, so that only trivial severity of poisoning will be recognizable [39].

### Taxonomy

The taxonomic status of the sea snakes is still under review and no general agreement exists at the moment. Traditionally sea snakes have been regarded as belonging to one family, Hydrophiidae, with *Laticauda* as the most primitive genus [27].

**Figure 7:**
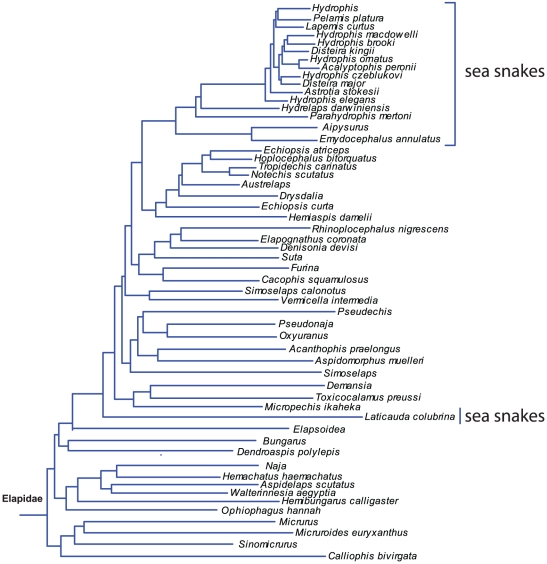
Sea snakes and their relationship to other Elapid snake genera. Note that the the “true” sea snakes (Hydrophiinae and Laticaudinae) are not closely related but nest within the terrestrial elapids. Generic names represent multiple species. The relationships among the many species of *Hydrophis* have not been resolved (see text for details). Modified after [127].

However, more recent results support the position that Laticaudinae and Hydrophiinae (true sea snakes) have evolved from different terrestrial elapids [34], [40]–[44] (**Figure 7**). The combined morphological and molecular results by Scanlon and Lee [44] support that sea kraits (*Laticauda*) and Solomon Islands elapids are basal to the remaining Australian terrestrial elapids and true sea snakes (Hydrophiinae). The Australian Elapids and true sea snakes include three main lineages: a large-bodied oviparous lineage, a small-bodied oviparous lineage, and a viviparous lineage which also includes the true sea snakes. The results by Castoe et al. indicate that *Laticauda* is closest to some Asiatic elapids [45]. However, these authors suggest to interpret these results cautiously because of long branch attraction. Sanders et al. [40] indicate that *Laticauda* is the sister group to all other hydrophiines (oxyuranines), which is also consistent with a classic morphological feature: all oxyuranines have reduced the choanal process of the palatine and lost the lateral process, permitting novel jaw movements [40], [46].

Some results indicate that true sea snakes (Hydrophiinae) can be separated into two totally different groups, indicating that sea snakes perhaps have evolved three times from terrestrial elapids [34], [47], [48]. However, this hypothesis is not supported by other recent results [40], [43], [44] and the latest molecular evidence suggests that the true sea snakes (Hydrophiinae) are a very young monophyletic group, perhaps only 8–13 Myr old [49] (**Figure 7**).

On the genus level at least three completely different taxonomic hypotheses exist, resulting in much confusion in both scientific and non-scientific publications. The most thorough work on sea snakes is by Smith, 1926, whose taxonomy has been used by most authors during the past 85 years. In 1972 McDowell, based on a few specimens from selected species, suggested separating the genus *Hydrophis* into 3 different subgenera based on morphological data. McDowell also changed some genera using the same arguments, which were not based on a phylogenetic analysis, but more on morphological similarity [50]. Kharin has suggested raising some of McDowells' species groups to genus level [23], [51]–[56], however, Kharin also did not produce any phylogenetic analysis for these groups. Recently, Wells [57] suggested changing the nomenclature in the Australian sea snake species also without any phylogenetic analysis, thus creating new problems for the layman to use valid generic names for the groups. A couple of papers using phylogenetic methods do not support the hypothesis by McDowell (1972) [43], [58], [59], [60]. Because of sea snakes' extremely venomous bite it is important to have correct taxonomic assignments and thus correct names because physicians often apply anti-venoms based on the species of snake. As long as no thorough phylogenetic analysis is available for the group, we suggest using Smith's [27] classification, with a few corrections that have been generally accepted [20]–[22], [29], [34].

Identifying sea snakes to species level is very difficult; especially the genus *Hydrophis* shows great intraspecific variation making it difficult to use only external characters for their identification. The ∼70 species recognized here follow Golay et al. [61] and David & Ineich [21] with a few new species added or resurrected [32], [62], [63]. A complete species list can be found in [1] when searching for the two subfamilies, Hydrophiinae and Laticaudinae, respectively.

At a higher taxonomic level sea snakes are the closest relatives of terrestrial elapids, which include some of the most venomous snakes in the world (e.g. brown snakes, taipan, death adder, cobra, krait, mambas) [40], [43]. Sea snakes or aquatic elapids and terrestrial elapids are collectively known as proteroglyphous snakes because of the position of the poison-fangs in the frontal part of the upper jaw (maxillary bone) [64].

### Feeding and breeding biology

Most sea snake species feed on fish that are close to the bottom or sedentary; a few prefer fish eggs (*Aipysurus eydouxii,* and the genus *Emydocephalus;* some specimens of *A. fuscus* also contained fish eggs) and at least *Aipysurus laevis*, *Enhydrina schistosa* and *Lapemis curtus* have been found with crustaceans and mollusks in their stomachs [58], [65]–[73]. The *Laticauda* group feed mostly on eels burrowed in sand at the bottom of the sea and in reef crevices [66], [72], [73]. Using sea snakes to capture undescribed eel species has been shown to be very effective because the eels are extremely secretive in their habits giving no chance to collect them using traditional methods [73].

All sea snakes produce living young (viviparous) except for the genus *Laticauda* which is egg laying (oviparous) [20], [74]. The annual reproductive cycles are synchronous between males and females and they reproduce every second year with a clutch size that increases with the size of the female [71], [75]–[83].

### By-catch and commercial use of sea snakes

Sea snakes are not only of interest because of their poison, but also in connection with the commercial exploitation of reptile skin, organs and meat. Some species are accessible in great numbers (e.g. *Laticauda spp*., *Lapemis spp*. and some *Hydrophis spp*.) and are not protected by CITES (Washington convention).

Since at least 1934 sea snake meat and skins have been used commercially in the Philippines [84], but also in Australia. In Japan, Taiwan, Thailand and Vietnam sea snakes have been collected commercially [38], [85]–[89]. Local protection of sea snakes has been necessary to stop over-exploitation in the Philippines. In Australia commercial sea snake fisheries and by-catch have been investigated during the past 15 years [77], [90]–[97]. However, most sea snake fisheries in the Indian Ocean and in the Pacific are not reported in the literature and are beyond control of the local governments.

### Species distribution and density

Many of the more than 60 species of sea snakes have a broad distribution in both the Indian Ocean and the Pacific Ocean (**Figure 6**). Species such as *Acalyptophis peronii, Aipysurus eydouxii*, *Astrotia stokesii, Enhydrina schistosa, Lapemis curtus, Laticauda colubrina, L. laticaudata* and many *Hydrophis* species have been collected in both the Asian and the Australian region [21], [22], [26], [27], [63], [98] and are abundant in these areas. Other species are much less well known and some species are only collected from very restricted areas and therefore much more vulnerable for change in the environments. One of the rarest species is *Hydrophis parviceps* which is known from only two specimens collected in a very limited area in the southern Vietnamese part of the South China Sea [99], [100]. *Hydrophis sibauensis* is a recently described species which has been collected more than 150 km into rivers of Borneo and is only known from 3 specimens [32]. This is also the case for another recently described species, *Hydrophis laboutei* which is only known from two specimens collected at Chesterfield Reefs (New Caledonia) [62]. Other species with a limited distribution are therefore highly vulnerable to all kinds of environmental changes, including *Aipysurus fuscus, A. apraefrontalis* and *A. foliosquama* which have only been collected in the north west part of Australian coral reefs. Recent surveys indicate that there has been a drastic decline of specimens in this areas over the last 10–15 years ([101] and pers.comm. Michael Guinea). *Hydrophis semperi* and *Laticauda crockeri,* both species only known from Lake Taal in the Philippines and Lake Te-Nggano in Rennell Island, respectively [30], [33], also represent species that have a very restricted distribution and therefore are highly vulnerable to changes in the environment.

Our own (ARR) investigations in the Gulf of Thailand and part of Borneo indicated that the sea snake fauna in this area has also declined. In the Gulf of Thailand, especially populations of species going up rivers have disappeared (e.g. *Hydrophis torquatus* and *H. klossi*), but also in Borneo the number of specimens collected previously in great numbers inside river mouths appears to have declined. Trawl fisheries in the Andaman Sea (e.g. around Phuket, Thailand) also got fewer specimens and the fishermen also mention that the size of sea snakes they got in former times was much larger than the ones they get now. A small survey in Cambodia (ARR) in the year 2000/2001 indicated similar results to those found for Borneo and Thailand.

### Conservation: What can we do to help the sea snakes to survive?

The main threat to sea snakes is the cultural indifference to conservation issues by locals and, as a consequence, their commercial exploitation. Only raising awareness may reduce this kind of threat. Another problem in trying to help the sea snakes to survive is the very limited knowledge on their biology, especially in Asia. It is very important to get much more information on the biology in formulating management plans. For the moment we still miss information on breeding cycles, by-catch and mortality, growth rates, population density, sexual maturity and taxonomy in most areas. The effects of the exploitation or/and by-catch on the sea snakes are almost unknown except from the Philippines and Australia [93], [95], [96]. Some populations may already be in danger of extinction. The only way to have a sustainable yield is by monitoring and controlling by-catch and commercial catch of sea snakes, giving local governments a chance to intervene before a catastrophic collapse of local populations occur. However, to limit exploitation of the most common species sustainable and to protect the endangered ones, we need to have much more biological focus on the group.

One cause for the disappearance of sea snake species from rivers is at least in part due to the great problems with the ongoing pollution in many rivers in both Asia and Australia. Also the information on breeding areas for most sea snake species is unknown, but there is no doubt that also habitat destructions (eg. mangrove clearings and water crafts) have a negative influence on sea snake populations.

A first step in sea snake conservation is to distinguish the many species from each other, which is not an easy task, and with the global decline of taxonomists it may be more important to focus initially on the entire group. The next step is to get more knowledge of sea snake biology and then to focus on the by-catch and management plan to protect the endangered species and harvest only the more common ones. Concerning the more global problems of pollution and habitat degradation, we have to put more pressure on politicians and hope that they will come up with solutions for the benefit of both humans and sea snakes.

## Other Snakes Found in Marine and Brackish Environments

**Figure 8:**
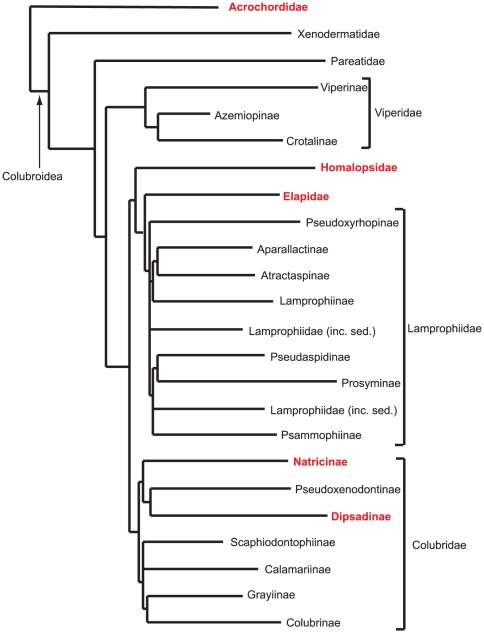
Several families of snakes have independently adapted to saltwater. Families with species that live in brackish or marine environments are shown in red.

Evolution is continually tinkering with snake populations that live in coastal areas adjacent to brackish and salt water environments. Besides the true sea snakes, many terrestrial and arboreal species have learned to exploit marine resources by foraging in the intertidal zone at low tide or from the branches of mangroves, while some freshwater species have adapted to life in brackish water, sometimes enter the ocean, or live there permanently (**Figure 8** **,** **Table 3**).

**Table 3:** Non-elapid snakes that use brackish water.

Genus	Total species	Species in brackish or marine water
*Acrochordus*	3	all (3)
*Agkistrodon*	4	1
*Bitia*	1	all (1)
*Cantoria*	1	all (1)
*Cerberus*	3	2+
*Crotaphopeltis*	6	1
*Djokoiskandarus*	1	all (1)
*Enhydris*	24	1
*Farancia*	2	all (2)
*Fordonia*	1	all (1)
*Gerarda*	1	all (1)
*Grayia*	4	1
*Helicops*	15	2+
*Hydrops*	3	1
*Liophis*	39	2+
*Myron*	3	all (3)
*Natrix*	4	3+
*Nerodia*	10	4+
*Regina*	4	1
*Stegonotus*	9	2+
*Storeria*	4	1
*Thamnophis*	31	2
*Tretanorhinus*	4	2

The list does not include arboreal and terrestrial species that use mangrove forests or terrestrial species that occasionally enter water, or forage in the intertidal zone. A “+” indicates there are probably more species in this genus that use brackish water.

All three living species of the ancient **Acrochordidae** use a combination of aquatic environments ranging from freshwater to sea water, and have probably been living in coastal ocean habitats for most of their 90.7 million year history [102]. Acrochordids, particularly *Acrochordus granulatus* have some of the most specialized morphology and physiology for life in saltwater, including the greatest capacity to store oxygen found in any vertebrate [103]. These are low metabolism snakes, feeding and reproducing infrequently and incapable of active swimming for more than a few minutes [104]. *Acrochordus arafurae* females ambush prey in deep water while males actively search for prey in shallow water [105]. They have a constriction-like behavior for holding prey between body loops during ingestion, and enlarged keels on their scales aid in holding slippery fish. Additionally each scale contains a mechanoreceptor that may be used to locate fish in turbid water [106].

**Figure 9:**
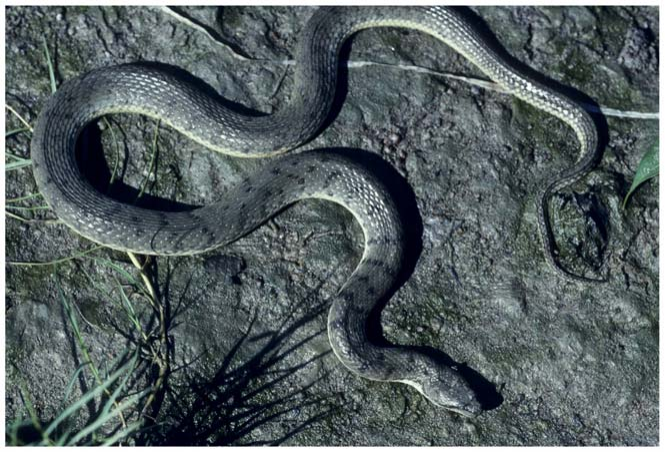
A homalopsid snake (*Cerberus rynchops*). See text for details.

The Asian and Australasian **Homalopsidae**, ironically sometimes called the “freshwater snakes,” have species that live in coastal habitats such as mangrove forests and salt marshes. Homalopsids inhabit life zones that range from the fossorial *Brachyorrhos albus* and the semi-terrestrial *Enhydris plumbea*, to *Erpeton tentaculatus* that is all but incapable of leaving their freshwater habitats, to species inhabiting coastal marine environments (*Bitia hydroides*, *Cantoria violacea*, *Myron* sp., *Fordonia leucobalia*, *Enhydris bennettii*). The most widespread and successful brackish water homalopsids are the bockadams (*Cerberus* sp) which are distributed from the vicinity of Mumbai, India in the east to Palau, Micronesia in the west, and range southward into the Indonesian Archipelago, New Guinea, and northern Australia (**Figure 6**). Like some of the *Hydrophis* and *Laticauda*, the Lake Buhi Bockadam (*Cerberus microlepis*) is a freshwater species, derived from its nearby salt water dwelling relative, *Cerberus rynchops* in the Philippines (**Figure 9**). While most of the aquatic homalopids are piscivorous, three species, all members of the same clade specialize in feeding on crustaceans (*Cantoria violacea*, *Fordonia leucobalia*, and *Gerarda prevostiana*) in near shore habitats [107]–[109].

An assemblage of mangrove homalopsids studied in Singapore [110] was dominated by the piscivorous Asian Bockadam, *Cerberus rynchops* (73% of total snakes), while the other species were crustacean specialists and less common. Gerard's Mud Snake, *Gerarda prevostiana* (16% of the total snakes) feeds exclusively on recently-molted crabs; while the Crab-eating Snake, *Fordonia leucobalia*, (10% of total snakes) specializes in feeding on hard-shell crustaceans. The most uncommon Singapore homalopsid was Cantor's Mud Snake, *Cantoria violacea*, (2% of total snakes) and it too has a specialized diet, feeding on *Alpheus* snapping shrimp (Decapoda, Alpheidae). The crustacean-eaters were often observed in association with mud lobster mounds constructed by *Thalassina anomala* (Decapoda: Thalassinidae). The snakes were nocturnal and active throughout the night. Gerard's Mud Snake increased activity during spring tides, but the other species did not. Three male Crab-eating snakes were tracked using radiotelemetry: they rarely moved and when they did it was for short distances (1.8 to 14.0 m). As might be expected for tropical aquatic snakes, the body temperatures were very stable (26.3–29.0°C) and consistently higher than the microhabitat temperatures. Two of the radio tracked crab-eating Snakes made extensive use of mud lobster mounds.

Snakes of the family **Natricidae** (or subfamily Natricinae) make up about 30 genera and 210 species [1]. They occur in both hemispheres and in both temperate and tropical environments, but they appear to have originated in Asia, and dispersed into Europe, and North America. Many of these snakes are semi-aquatic and some inhabit brackish water. In North Africa and Europe at least three species use coastal habitats: the Viperine Snake, *Natrix maura*, the Grass Snake, *N. natrix sicula*, and the Dice Snake, *N. tessellata* [111]–[113]. North American brackish water natricids use mangrove and salt marsh habitats include the Mangrove Water Snake, *Nerodia clarkii,* in the southeastern USA, and the Baja Garter Snakes, *Thamnophis valida*. Both these species are piscivorous and like some freshwater natricids, *Nerodia clarkii* juveniles lure prey with their tongue [114]. The Salt Marsh Brown Snake, *Storeria dekayi limnectes* is less aquatic than the previous species, and it feeds on soft bodied invertebrates [115].

In the Neotropics, the family **Dipsadidae** (or subfamily Dipsadinae) has more than 92 genera and >700 species [1], a few of which use brackish and salt water habitats (*Helicops*, *Hydrops*, *Liophis*, and *Tretanorhinus*) to varying degrees. For the most part these snakes are poorly known and their brackish water habits are known only from anecdotal observations [116]. Similarly, at least two aquatic African snakes, of uncertain lineages (*Grayia smythii* and *Crotaphopeltis hotamboeia*) occur in the brackish water of mangrove forests as well as freshwater [117], [118].

There is another group of snakes that use marine resources that is worthy of note. These species forage in mangroves, salt marshes, and intertidal zone without actually spending much time in the water. The most specialized is perhaps the Burmese Vine Snake, *Ahaetulla fronticincta* (family **Colubridae**) which hunts gobies from branches over hanging the water [28]. The Southeast Asian Mangrove Pitviper, *Cryptelytrops purpureomaculatus* (**Viperidae**) also hunts from the mangrove branches but its diet is unknown. There are others, for the most part habitat generalists with populations in or adjacent to marine habitats and include *Python molurus*, *Python reticulatus* (**Pythonidae**), *Ophiophagus hannah* (**Elapidae**), and *Boiga dendrophilia* (**Colubridae**) – all medium to large snakes that forage for food in coastal habitats. Other snakes forage in the intertidal zone: *Coluber anthonyi* (Colubridae); *Crotalus mitchelli* and *Crotalus muertensis* (family **Viperidae**, subfamily Crotalinae).

Thus, of 34 lineages of snakes (families and subfamilies), four (Acrochordidae, Homalopsidae, Dipsadidae, and Elapidae) contain most of the species adapted for marine environments, while other clades have relatively few, or no, species adapted for the saline water. As more herpetologists investigate mangroves and salt marshes a more complete picture of brackish water snakes will emerge.

## Marine Iguanas

Lizards are the most speciose and diverse group of reptiles, with almost 5,500 species (60% of all reptiles) [1]. Nevertheless, only a few species have ventured into the oceans. The marine iguanas of the Galapagos Islands are the most aquatic of the lizards, but bask and reproduce on land and are subject to terrestrial predators.

The Galapagos archipelago arose through volcanic activity 960 km off the coast of Ecuador. Lizards and other animals probably reached the island on rafts of vegetation washed down the rivers of western South America. The rafts may have included juveniles, adults, or eggs. The Humboldt and El Niño currents would allow for such rafting to originate from the western coast of South or Central America. Other species, such as rats, have been introduced by humans only about 100 years ago.

Besides seven species of smaller iguanids of the genus *Microlophus* four large species of iguanas inhabit the Galapagos islands [119]: three species of land iguanas (genus *Conolophus*) and the related marine iguanas (*Amblyrhynchus cristatus*). Because the land iguanas are endemic to only a few islands, they are threatened by predation of introduced mammals. However, recent measures to control and protect their habitats seem to have stabilized their populations. In addition to these anthropogenic factors, the archipelago is subject to strong seasonal and annual variations in environmental conditions, so that a combination of factors could turn out to be detrimental.

The marine iguana, *Amblyrhynchus cristatus*, and the land iguanas of the genus *Conolophus* are all about 1.2 m in length. *Amblyrhynchus* inhabits virtually all islands of the archipelago, given its ability to disperse to various islands. As a result, they are less prone to extinction.

Although land and marine iguanas are very different in appearance, they are closely related. In fact, several instances of hybridization between the two genera have been reported [120], [121]. Hence it is likely that they both evolved relatively recently from a land iguana that came from the South American mainland.

Marine iguanas feed exclusively on marine plants. While they spent a considerable time in the water foraging, they have not completely adapted to marine life. For instance, they still have to nest on land and also bask on land to reach their optimal body temperature which rapidly declines in the rather cool ocean water. Nevertheless, considerable selection pressure has resulted in several adaptations to their marine lifestyle such as a flattened tail and limited webbing of all four feet, supporting swimming. Powerful claws help them to hold on to rocks in the heavy sea. Marine iguanas also have reduced the number of heartbeats per minute from about 43 on land to 7 to 9 while diving, as do several other reptiles [122]. Finally, both *Conolophus* and *Amblyrhynchus* possess nasal salt glands, similar to those found in other reptiles that have high dietary salt intakes [122]. Interestingly, neither species has the capacity to produce hyperosmotic urine. Thus, the marine iguana has the highest known extracloacal excretion rate of Na, Cl, and K of any reptile [123].
